# Exosomes in atherosclerosis: Convergence on macrophages

**DOI:** 10.7150/ijbs.71862

**Published:** 2022-05-01

**Authors:** Kaiying Yang, Qi Xiao, Mengying Niu, Xudong Pan, Xiaoyan Zhu

**Affiliations:** 1 Department of Neurology, The Affiliated Hospital of Qingdao University, Qingdao, China,; 2 Department of Critical Care Medicine, The Affiliated Hospital of Qingdao Universality, Qingdao, China

**Keywords:** Atherosclerosis, Macrophage, Exosomes, Biomaterials, Extracellular vesicles

## Abstract

As the primary cells of atherosclerotic plaques, macrophages play a central role in the occurrence and progression of atherosclerosis (AS). In recent years, macrophages have received extensive attention as therapeutic targets. Exosomes, as natural nanoparticles, have high biocompatibility and strong targeting ability and have been widely studied as imaging agents and drug carriers. Studies on the relationship between atherosclerotic macrophages and exosomes have been focused on for the past few years. Nevertheless, no complex review has been undertaken in this area. In this review, we summarize in detail the role of macrophages in atherosclerosis, especially their plasticity and phenotypic and distributional heterogeneity. Based on the high correlation between macrophages and the pathological process of atherosclerosis, as well as the targeting of exosomes, we further review the clinical application of targeting macrophage-associated exosomes. We focus on the role of macrophage-associated exosomes in the phenotypic transformation of cells in atherosclerosis, providing a new idea for the clinical application of targeting macrophage-associated exosomes. Finally, we specifically summarize and prospect the diagnosis of macrophage-associated exosomes, such as imaging agent delivery, biomarkers and therapeutic strategies.

## Introduction

Atherosclerosis, which can be traced back to 4000 years ago, refers to the thickening of the intima caused by lipid and fibrous substances deposition in the innermost layer of the large and medium arteries 1, 2. As a plaque grows, the lumen becomes clogged, preventing blood flow from reaching the donor area, leading to distal tissue ischemia. In addition, rupture of vulnerable plaques with a peculiarity of a larger lipid core but thinner fibrous cap can activate clotting pathways, forming thrombi that quickly block the lumen and cause acute symptoms 3. Atherosclerosis leads to cardiovascular and cerebrovascular diseases, such as acute coronary syndrome and stroke, which are the leading cause of death worldwide, according to the* Global Burden of Disease* in 2016 4.

The pathological mechanisms of atherosclerosis, particularly chronic inflammation and immune responses, have been extensively studied 5-9. Classical monocytes that circulate in blood can interact with endothelial cells through adhesion molecules and enter the intima under the action of chemokines. Monocytes entering the intima differentiate into macrophages, the majority of immune cells in atherosclerotic plaques, when exposed to the cytokine-enriched microenvironment 10-13.

As the key cells in atherosclerosis, the heterogeneity of the phenotype and distribution of macrophages has long been widely studied. Based on the discrepant metabolism of arginine in macrophages of the Th1 strain (C57BL/6) and Th2 strain (Balb/C), Mills first proposed the M1 and M2 dichotomy. Subsequently, the development of single-cell technology has improved our understanding of the heterogeneity of macrophages 14-17. Notably, a series of phenotypic transformations, such as proliferation, apoptosis, and polarization of macrophages, are closely associated with changes in microenvironmental signals 18, 19, so targeting the plaque microenvironment of macrophages could be a potential therapeutic strategy. As a bridge of intercellular communication, exosomes have been extensively studied in the proliferation and metastasis of tumor cells 20-22. Therefore, the role of various biological signals carried by exosomes in the plaque microenvironment may be a potential target for the transformation of macrophage phenotypes into beneficial directions.

Exosomes with a size of ~40 to 160 nm are a type of extracellular vehicles (EVs) that can be set free by all cells, including eukaryotes and prokaryotes. Through a series of invaginations of the plasma membrane, endosomes form multivesicular bodies (MVBs) and acquire various contents simultaneously 20, 23, 24. Exosomes derived from macrophages have been shown to play a regulatory role in inflammation, and their special role in various diseases, such as cancer, cardiovascular disease, atherosclerosis, and autoimmune diseases, as well as their potential clinical application as drug transport, diagnosis, and treatment, has been confirmed by numerous studies 25-28.

Traditional treatment methods (lipid-lowering, systemic anti-inflammatory therapy) do not achieve completely satisfactory results, and many studies are trying to find a new effective treatment method 29. The key role of macrophages in lipid uptake and inflammation development, as well as exosomes that act as bioinformation carriers with high biocompatibility, contribute to the realization of macrophage-targeted therapy to solve the disadvantages of traditional therapies 30, 31. Meanwhile, surface-functionalized exosomes *in vivo* tracing techniques and their applications in *in vivo* imaging and targeted drug delivery have been reported 32-34. Therefore, revealing the relationship between exosomes and macrophages can offer new insights into the diagnosis and treatment of atherosclerosis based on macrophages.

In this review, we provide a novel perspective for the application of exosomes in atherosclerosis. We introduce the central role of macrophages in atherosclerosis in detail, focusing on the heterogeneity of macrophages, including phenotype, distribution, and function. Furthermore, the role of macrophage-associated exosomes (including exosomes derived from macrophages and exosomes internalized by macrophages) in the phenotypic transformation of plaque cells will be emphatically discussed. At the same time, its potential applications in clinical diagnosis and treatment will be stated. Although exosomes have not yet been used in the clinical treatment of atherosclerosis, we believe that the successful application of exosomes in cancer diagnosis and treatment could accelerate the clinical transformation of exosomes therapy that focuses on the atherosclerotic macrophage.

## The central role of macrophages in atherosclerosis

### Macrophage: initiation and progression of atherosclerosis

**Figure 1:**
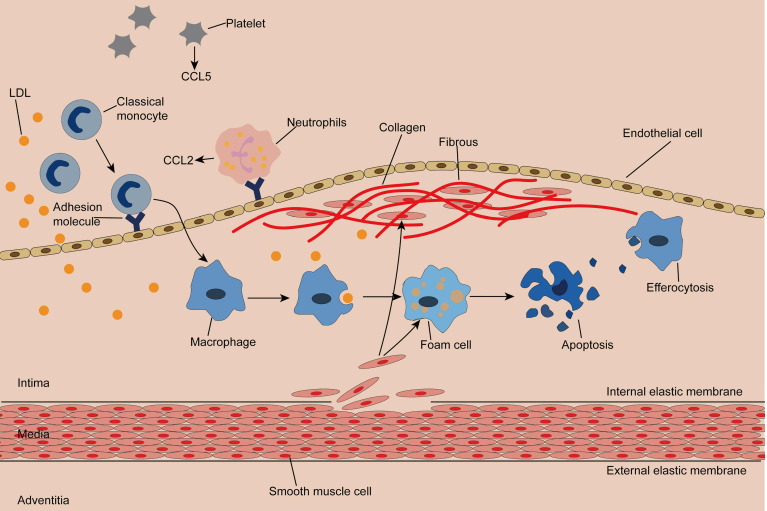
** Initiation and progression of atherosclerosis** Monocytes enter the damaged subendothelial under the action of chemokines and adhesion molecules and differentiate into macrophages. Macrophages engulf lipid particles in the endothelium and become foam cells, forming streak, an early lesion of atherosclerosis. Smooth muscle cells acquire macrophage properties and phagocytose lipids, constituting another source of foam cells. Smooth muscle cells produce collagen to form fibrous caps. The toxicity of cholesterol leads to the apoptosis of foam cells, and macrophages remove the apoptotic cells by efferocytosis. Inadequate efferocytosis enlarged lipid cores.

The normal artery wall is made up of three layers: the adventitia, the media, and the intima, where plaques occur 4, 35, 36. (Figure 1) Classical monocytes are recruited to the intima in response to endothelial cell activation due to hypertension, hyperglycemia, or dyslipidemia 4, 37. Recruitment of monocytes depends on the cytokine microenvironment composed of chemokines, cytokines, and adhesion molecules. Activated platelets release C-C motif chemokine 5 (CCL5), which promotes the adhesion of monocytes and neutrophils to endothelial cells. Neutrophils attached to endothelial cells continue to secrete chemotactic granular proteins, such as antimicrobial peptides, cathepsin G, and CCL2. Activated smooth muscle cells secrete CCL2 and CCL5, complementing the microenvironment and facilitating monocyte infiltration 7. Of note, exosomes isolated from IFN-α-stimulated monocytes significantly increased the endothelial cell adhesion molecule ICAM-1 and promoted monocyte recruitment by activating NF-κB 38. Once inside the intima, monocytes differentiate into macrophages and remove LDL particles retained in the intima by expressing scavenger receptors (SR), forming foam cells with droplet filling. At this point, a portion of cholesterol is reserved in macrophages and thus mitigates cholesterol-induced cytotoxicity, while superfluous cholesterol is transported out of the macrophage by membrane lipid translocases known as ATP-binding cassette (ABC) transporters 39, 40.

Cholesterol crystal accumulation activates the NF-κB signaling pathway, which mediates activation of the NLRP3 inflammasome and production of proinflammatory cytokines, both of which increase inflammation, while enlarged inflammation leads to recruitment of more monocytes 41. In addition, intimal thickening is caused by the proliferation of smooth muscle cells inherent in and migrating from the middle layer, as well as an increase in extracellular matrix synthesis and macrophages 42, 43. Smooth muscle cells produce collagen, which is inhibited by IFN-γ and proinflammatory macrophages. Notably, proinflammatory macrophages can also produce matrix metalloproteinases, leading to thinning of the fibrous cap 44, 45. As the disease progresses, dying cells, dead cells, and lipid particles form a necrotic core that gradually expands. The thin fibrous cap and large necrotic core constitute a typically vulnerable plaque. When the fibrous cap ruptures, the clotting factors in blood flow encounter the tissue factors in the plaque, triggering clotting and resulting in thrombosis and a series of cardiovascular adverse events 46, 47. Therefore, identifying susceptible individuals and stabilizing vulnerable plaques are of great importance to prevent atherosclerotic complications.

## Heterogeneity of macrophages: phenotype and distribution in plaques

### Major macrophage subsets in atherosclerotic plaques

There are two main sources of macrophages in atherosclerotic plaques: monocytes that penetrate the endothelium and tissue-resident macrophages. Growing evidence indicates that the origin of macrophages may ultimately determine cell function^48^. In mice, monocytes can be divided into two types according to the Ly6C expression level, while in humans, this heterogeneity is reflected in the expression levels of CD14 and CD16. The subset with high Ly6C expression corresponds to classical monocytes (CD14^++^/CD16^_^ subtype) with the highest proportion in humans, while Ly6C^lo^ is similar to the nonclassical CD14^+^/CD16^++^ subtype. Unlike mice, humans also have an intermediate CD14^++^ /CD16^+^ subtype 49-52, a transitional state between classical and nonclassical subpopulations, which can be explained by surface markers 52.

Likewise, macrophages can transform into different subgroups depending on the stimulation. Gordon described M1 macrophages as classically activated macrophages differentiated by Toll-like receptor (TLR)-mediated signaling or cytokine stimulation induced by IFN-γ and TNF, while alternatively activated macrophages stimulated by Th2 cytokines, such as IL-4 and IL-13, were synonymous with M2 macrophages^53^. M1 macrophages are usually derived from classical monocytes, while M2 macrophages are derived from non-classical monocytes 54. M1 macrophages can release chemotaxis and the proinflammatory cytokines CCL2 (MCP-1), CCL3, IL-1β, IL-6, IL-12, IL-23 and TNF to trigger inflammatory responses and secrete reactive oxygen intermediates (ROIs), nitric oxide (NO) and lysosomal enzymes to kill and clear pathogens, while ROS induce tissue damage, which is not conducive to wound repair 55. M2 macrophages, on the other hand, secrete anti-inflammatory cytokines, such as IL-10 and TGF-β, to counter the inflammatory response caused by M1 to reduce tissue damage. In addition, alternating activation of macrophages can secrete platelet-derived growth factors and fibroblast growth factors to participate in injury repair and fibrosis 53, 56, 57. This dichotomy model is based on extreme phenotypes observed *in vitro*, but the *in vivo* environment is complex, and different stimuli can induce different subgroups. Apparently, this classification seems to be oversimplified in atherosclerotic plaques.

**Figure 2:**
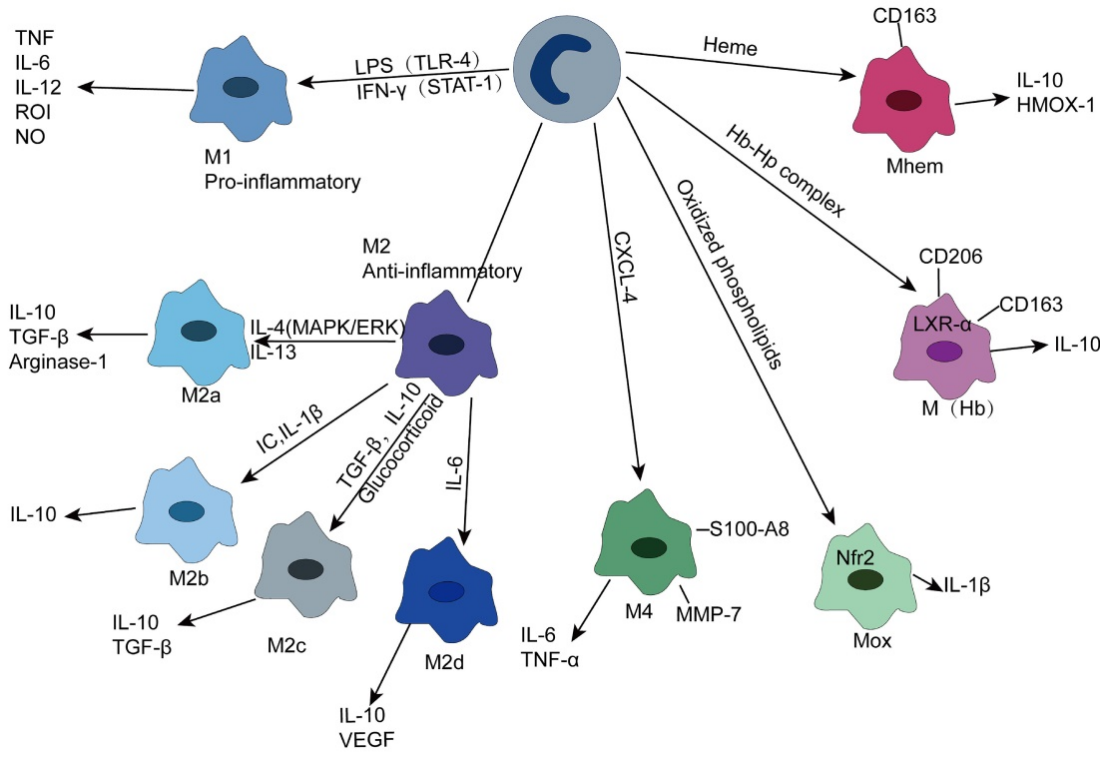
Macrophage subtype within the plaque

Due to different activation signals, M2 macrophages can express different molecular markers and secrete different cytokines (Figure 2), so they can be divided into four subgroups.

(1) M2a—M2a macrophages are induced by IL-4 and IL-13 and secrete anti-inflammatory cytokines, such as IL-10 and TGF-β, which are involved in anti-inflammatory processes and injury repair. In addition, they can express high levels of mannose receptor (MR ,CD206). It is noteworthy that M2a secretes arginase-1, which can decompose L-arginine into L-ornithine and L-proline, a process different from the L-arginine decomposition of M1 type 53, 58, 59.

(2) M2b—M2b macrophages can be induced by immune complex, IL-β, and TLR signaling to differentiate and secrete high levels of IL-10, which plays an immunomodulatory role in atherosclerotic plaques. Unlike M2a and M2c, M2b also produces high levels of proinflammatory cytokines, such as IL-1, IL-6, and TNF 55, 60.

(3) M2c—M2c is the most prominent anti-inflammatory subtype triggered by IL-10, TGF-β, and glucocorticoids and produces IL10, TGF-β, and pentraxin 3 (PTX3) 61.

(4) M2d—This polarization process can be mediated by IL-6 and adenosine A2A receptor, which can produce IL-10 and vascular endothelial growth factor that can participate in angiogenesis. It should be noted that M2d, unlike M2a, M2b and M2c, does not express mannose receptors 62.

In addition to M1 and M2 types, other macrophage subsets exist in atherosclerotic plaques. Kadl et al. demonstrated for the first time that oxidized phospholipids, which are abundant in mouse atherosclerotic lesions, induce Mox macrophages lacking phagocytosis. This type is mainly present in mice. The formation of this group of macrophages depends on the transcription factor Nrf2, which plays a vital role in promoting disease progression and inflammation due to its ability to secrete IL-1β and cyclooxygenase 2 63. M4 macrophages are induced by the platelet chemotactic protein CXCL-4 and lack phagocytosis. Notably, CXCL-4 can downregulate CD163 expression, while CD163^-^ macrophages are unable to respond to Hb-Hp complex exposure by upregulating heme oxygenase-1 (HMOX-1), an enzyme that has a protective effect on atherosclerosis 55, 56, 64.

M(Hb) is a macrophage with high expression of mannose receptor (CD206) and scavenger receptor (CD163). Since CD163 can absorb the Hb-Hp complex released after RBC destruction, M(Hb) is mainly distributed in the plaque neovascularization prone site. Clearance of free hemoglobin resulted in increased transferrin expression and decreased intracellular iron and reactive oxygen species. Increased liver X receptor (LXR) α expression leads to cholesterol efflux, ultimately leading to the characterization of low lipid storage of M(Hb) 65. In addition, heme-stimulated monocytes can differentiate into Mhem, a macrophage phenotype that is resistant to lipid uptake and inhibits oxidative stress, depending on the AMPK -- ATF-1 -- LXRα -- LXRβ -- ABCA1 axis and the simpler ATF-1 -- HOMX-1 axis 66.

### Other classifications

Single-cell RNA sequencing and other techniques enable us to have a more comprehensive understanding of the phenotype and function of macrophages. Cole et al*.* combined mass spectrometry and clustering algorithms and found that there were 20 myeloid cell clusters and at least 5 macrophage subpopulations in the atherosclerotic aorta of mice, in order of proportion: (1) CD206^+^CD169^+^CD209^-^ (2) CD206^+^CD169^+^CD209^+^ (3) CD11c^+^ (4) CD206^LO-int^ (5) F4/80^high^ CD11b^high^. The proportion of proinflammatory CD11c^+^ was increased in Apoe^-/-^ mice fed a high-fat diet, suggesting that a high-fat diet promotes the inflammatory progression of atherosclerosis 67.

In a comprehensive meta-analysis of 9 single-cell RNA sequencing and 2 mass cytometry datasets, Zernecke et al. identified four known macrophage subpopulations in the aorta of atherosclerotic mice: resident-like cells, inflammatory cells, interferon-induced cells, and foam macrophages from Trem2 (trigger receptor 2 expressed on bone marrow cells). In addition, a new subpopulation of macrophages similar to cavity-type macrophages was identified 68. Resident-like macrophages express genes previously related to resident aortic macrophages, such as Lyve1 and Mrc1, as well as Pf4 genes originally thought to be platelet-specific 69. As the name suggests, inflammatory macrophages highly express proinflammatory genes, including the chemokines Cxcl1, Cxcl2, Ccl2, Ccl3, and Ccl4 and the inflammatory cytokines Il-1b and Tnf 70, 71. In contrast, foamy Trem2 macrophages express low levels of inflammatory genes and high levels of MMP12, MMP14, and lipid conforming markers, such as Abcg1, Trem2, and Fabp4 71. IFN-inducible macrophages (IFNICs) form a small cluster with a typical IFN-Ⅰ response, expressing Ifit3, Irf7, and Isg15 70, 72. Finally, with regard to cavity macrophages, a small cell population shows similarity to monocyte-derived peritoneal small macrophages due to its expression of genes encoding Cd226, Itgax (CD11c), Ccr2, Retnla, and MHC-II 73. Accurately identifying the role of aortic macrophage subpopulations in regulating atherosclerosis, as well as their function and interaction with other immune cells, such as T cells and DCs, is critical to understanding their relevance to the progression of atherosclerosis.

### Spatial distribution of macrophages in plaques

Different permutations and combinations of stimuli induce a variety of macrophage subsets. The lesions of atherosclerosis are not constant, so the stimulation signals also evolve dynamically according to space-time, which results in a heterogeneity of the distribution of macrophages in plaques 17. M1 and M2 macrophages run through the progression of human atherosclerosis, while in mice, the M2 type is considered mainly present in the early stage of the disease, while M1 is the primary macrophage in advanced plaques 74, 75. Due to its proinflammatory properties, M1 macrophages are considered to be the dominant player in plaque vulnerability, mainly located in a plaque vulnerable area—the shoulder 76. IL-4-mediated M2 polarization via activation of STAT6, due to its high phagocytosis ability, can remove apoptotic cells and facilitate the regression of inflammation 77. Unstable plaques are the main cause of adverse cardiovascular events, and maintaining existing plaque stabilization can be used as a treatment strategy for the prevention of myocardial infarction and stroke. The goal is to find a balance between proinflammatory and anti-inflammatory factors, namely, to find a balance between M1 and M2, which can be observed in relatively stable plaques covered with fibrous caps with the same number of two types of cells 17. In addition, the progression of atherosclerosis can lead to increased local hypoxia and, as a compensatory mechanism, the formation of leaky new blood vessels within the plaque, which is not conducive to plaque stability and can lead to plaque bleeding. M (Hb) and Mhem are found in neovascular-rich regions because they express CD163 of the ingestible Hb-Hp complex. At the same time, their ability to inhibit oxidative stress and promote cholesterol effusion suggests that their effects on atherosclerosis are positive 78, 79. M4 is thought to promote atherosclerosis, and due to its expression of metalloproteinase MMP7 and calcium-binding protein S100-A8, the presence of M4 is not conducive to plaque stability 56.

### Role of macrophage in atherosclerosis

As the central cells of atherosclerosis, macrophages can influence plaque progression in several ways, such as maintaining lipid homeostasis, opposing effects on inflammation, efferocytosis, and influencing plaque stability by producing MMP.

Lipid deposition in the intima is an important event in atherosclerosis 4. Macrophages remove cholesterol by expressing scavenger receptors and become foam cells, forming lipid streaks in early atherosclerotic lesions 29. Hyperlipidemia can promote the accumulation of macrophages in the intima, mainly by fostering hematopoiesis (including bone marrow hematopoiesis and spleen hematopoiesis) to increase the number of macrophages from monocytes and promote the proliferation of tissue-resident macrophages 42, 49. Part of oxidized cholesterol is phagocytic and stored in cells to prevent cholesterol-induced cytotoxicity, while excess cholesterol spillover is mediated by PPARγ- and LXRα -induced expression of ATP-binding box transporters ABCA1 and ABCG1 39, 40, 80, 81. It is noteworthy that ApoE, as a polar regulator of monocyte and macrophage, regulates lipid-induced hematopoietic stem/multipotential progenitor cell (HSPC) proliferation via ABCA-1/ABCG-1 82. LXRα agonists have been shown to improve atherosclerosis in animal models 83, 84. Hb-associated subsets M(Hb) and Mhem mediate low levels of lipid accumulation through a high expression of LXRα and low expression of lipid clearance-related SR 65. In contrast, M2a expressed low levels of LXRα, resulting in a lower cholesterol efflux capacity 78. When cholesterol builds up too much, this outflow ability is impaired, making it difficult to maintain cholesterol balance and thus exacerbating atherosclerosis.

Based on histopathological and anatomical pathological observations and deductive reasoning, Rudolf Virchow described inflammation in atherosclerotic plaques in the mid-19th century 85-87. Since then, an increasing number of studies have revealed the key role of inflammation in atherosclerosis. Treatment with statins and PCSK9 inhibitors to lower LDL did not reduce the risk of cardiovascular adverse events. However, the CANTOS trial (blocking IL-1β with canakinumab) and the COLCOT trial (colchicine treatment) showed a reduced risk of cardiovascular events 88, 89. Therefore, treatment strategies that target inflammation are important. The proportion of macrophages that promote or deactivate inflammation determines whether atherosclerosis progresses. ApoB-LPS activates endothelial cells, upregulates the expression of adhesion molecules (P-selectin, VCAM-1, and ICAM-1), and secretes chemokines inducing monocytes to recruit, infiltrate, and differentiate into macrophages 29, 90. Proinflammatory macrophages can secrete a series of inflammatory mediators, such as IL-1β, IL-6, IL-12, and TNF-α, to increase inflammation, which in turn attracts more monocytes 91. In contrast, anti-inflammatory macrophages, usually M2 macrophages, secrete high levels of IL-10 and TGF-β, which subside inflammation. Expanding the anti-inflammatory effect of macrophages is expected to prevent the progression of lesions and induce the degeneration of atherosclerosis. Based on the development of nanomedicine (especially exosomes), it is possible to target macrophages to inhibit inflammation therapy 29.

Plaque stability is an important determinant of cardiovascular events. Vulnerable plaques are characterized by thin fibrous caps and large lipid cores. The stabilizing effect of macrophages on plaques is not the same direction: macrophages can reduce the necrotic core to increase stability or thin the fibrous cap to become plaques prone to rupture 92. Efferocytosis refers to the process how phagocytes remove apoptotic cells and their fragments 93. In atherosclerosis, M2 macrophages with stronger phagocytosis remove apoptotic cells through high expression of MerTK to reduce the necrotic core and increase plaque stability 94. Even so, a lack of efferocytosis can lead to secondary necrosis and promote the formation of necrotic nuclei 93, 95. VSCMs can produce collagen, which can be inhibited by proinflammatory macrophages 96. In addition, M1 macrophages have high expression of metalloproteinases (MMP-1, MMP-3, MMP-10, MMP-12, MMP-4, and MMP-25), both of which result in decreased fibrous cap thickness 97. In addition, microcalcifications also increase the risk of plaque rupture, while large calcifications form organized structures that stabilize atherosclerotic plaques 98-100.

## Formation of macrophage-derived exosomes and internalization of exogenous exosomes

Due to the defects of traditional anti-inflammatory and lipid-lowering therapy, targeted therapy is urgently needed to improve efficacy and reduce adverse reactions. With the deepening of the research on macrophages and the revelation of the inflammatory regulation of macrophage-derived exosomes 101, a new anti-atherosclerosis therapy has emerged, targeting macrophage-associated exosomes.

Exosome formation involves three steps: double invagination of the plasma membrane, intracellular multivesicular bodies (MVBs), and formation and release of intraluminal vesicles (ILVs). This is true in most cells, including macrophages. Specifically, membrane invagination of macrophages forms an early sorting endosome (ESE) containing many proteins and extracellular matrix, which then matures into a late sorting endosome (LSE) and undergoes a second invagination (endosomal limiting membrane) to form MVBs containing multiple intraluminal vesicles (ILVs). MVBs can fuse with lysosomes or autophagosomes and be degraded, and can fuse with plasma membranes to release ILVs, commonly known as exosomes 20, 25, 102, 103. Multiple protein complexes, including GTPase Rab, Sytenin-1, TSG101 (tumor susceptibility gene 101), ALIX (apoptosis linked gene 2 interaction protein X), polymer-1, ESCRT (endosomal classification complex required for transport) proteins, phospholipids, tetraspanins, ceramides, sphingomyelinases, and SNARE (soluble Malay Imide sensitive factor (NSF) attachment protein receptor), participate in and restrict this process. However the specific mechanism still needs to be further explored 20, 104. Similar to macrophage polarization, the formation and release of exosomes are also regulated by the extracellular environment. Li et al. demonstrated that LPS-induced exosome release from macrophages was internalized by adjacent macrophages to promote TNF-α expression, which was inhibited by interleukin (IL-25) secreted by lung epithelial cells by downregulating the expression of Rab27a and Rab27b in macrophages 105. In addition to LPS, the classical activator of Toll-like receptors, Ibanez et al. demonstrated for the first time that alcohol and lipid raft interactions also trigger TLR4 signaling responses, thereby increasing astrocyte-derived EV release 106.

Exosomes can be secreted by various cells into various biological fluids, such as serum, breast milk, urine, and saliva, and mediate intracellular communication, thus affecting the development of disease. The transport and distribution of exosomes in body fluids and tissues are influenced by their size, cell origin, route of administration, and composition of exosomes 20. Munagala et al. demonstrated that milk exosomes have great potential as drug carriers for hydrophilic and lipophilic agents, including chemotherapy drugs. This nanotechnology can ameliorate the limitations associated with poor oral bioavailability of chemotherapeutic agents and reduce the total dose administered to reduce toxicity. In addition, exosomes from milk also have the characteristics of targeted ligand binding and cross-species tolerance, which can reduce systemic adverse reactions and adverse immune responses caused by missed targets 107. When reaching the target cell, exosomes can trigger signals by interacting directly with extracellular receptors, can be ingested by directly fusing with the plasma membrane, or can be internalized through the following five pathways: 1) clathrin-mediated endocytosis 2) lipid raft-mediated endocytosis 3) caveolin-mediated endocytosis 4) phagocytosis 5) micropinocytosis 108.

## Macrophage-associated exosomes in arteriosclerosis

### Effects of macrophage-derived exosomes internalized by other cells

**Figure 3:**
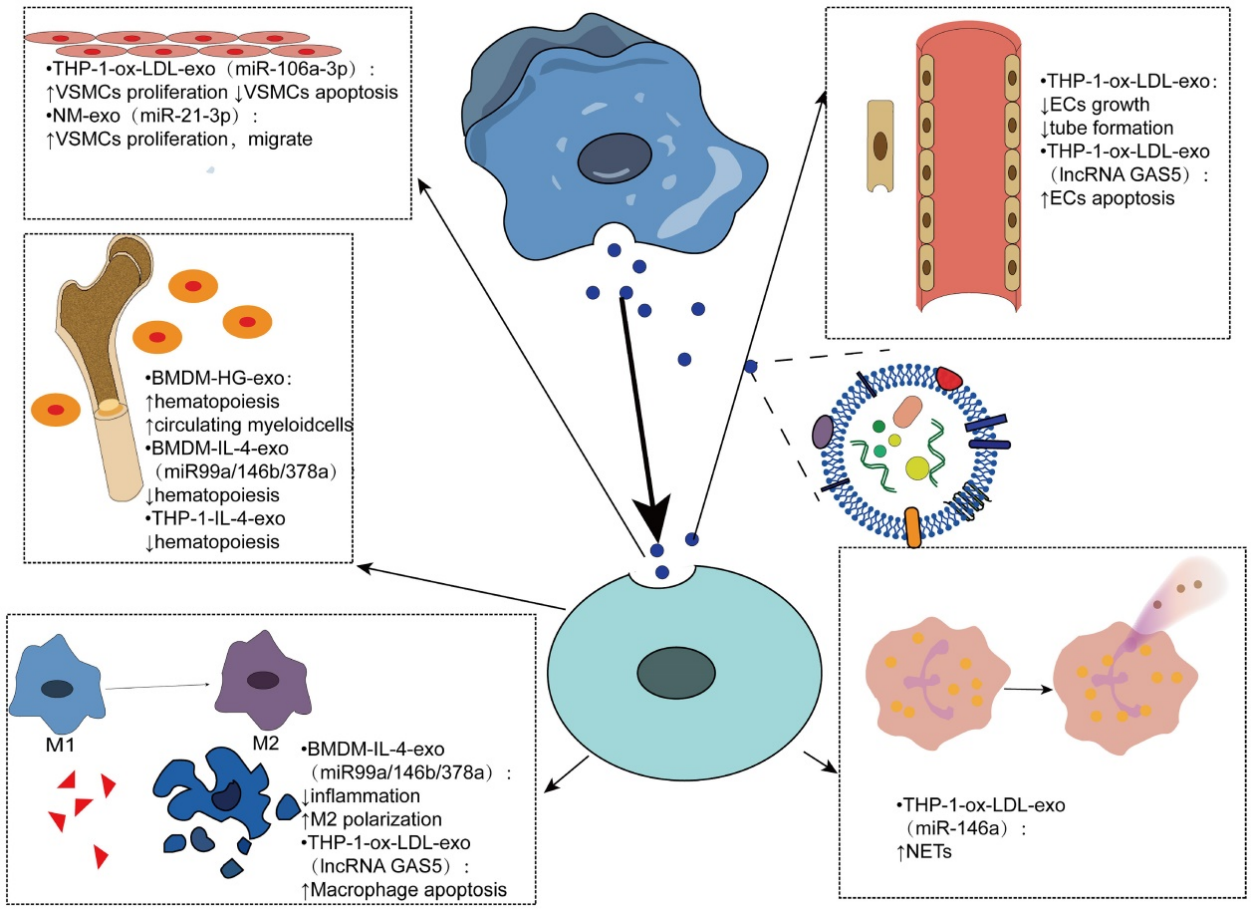
** Effects of macrophage-derived exosomes being internalized by other cells** Macrophage-derived exosomes mediate intercellular communication in various cells such as vascular smooth muscle cells, endothelial cells, myeloid cells, and neutrophils. By carrying contents, mainly microRNAs, mediates various phenotypic transformations, such as cell proliferation, apoptosis, hematopoiesis, polarization, and neutrophil traps.

Exosomes derived from macrophages play an important role in atherosclerosis by mediating cell-cell communication, mainly in regulating cell proliferation, apoptosis, inflammatory response, hematopoiesis and other aspects (Figure 3). In an experiment using bone marrow-derived macrophage exosomes (BMDM-HG-exos) produced in a simulated environment of human hyperglycemia, Bouchareychas et al. observed metabolic rearrangement and increased cell proliferation of BMDMs exposed to BMDM-HG-exos. When these exosomes were injected into apoe^-/-^ mice, hematopoietic expansion and an increase in the number of myeloid cells were observed. Furthermore, they found that BMDM-HG-exos amplify HSPC expansion by decreasing the expression of Abca1 mRNA in recipient BMDMs, which reduces cholesterol efflux 109-112. Another study by the same team demonstrated that BMDM-exos contain the anti-inflammatory microRNA 99a/146b/378a, which inhibits inflammation by targeting the NF-κB and TNF-α signaling pathways, providing a new direction for anti-inflammatory therapy for atherosclerosis 101. THP1-IL-4-exo regulates energy metabolism by regulating microRNA levels, and *in vivo* injection of THP1-IL-4-exo into two different mice with lipid metabolism disorders has been shown to reduce hemopoiesis and myelopoiesis 113.

Exosomes have been shown to regulate the biological function of VSMCs and promote the progression of AS by transferring miRNAs or proteins. Ox-LDL-induced elevation of miR-106a-3p in THP-1 macrophage exosomes and its binding with CASP9, a gene that encodes caspase-9 114, inhibits the caspase signaling pathway in VSMCs, resulting in increased proliferation and decreased apoptosis of VSMCs 115. The formation of neutrophil traps, also known as NETosis 116, can be triggered by cholesterol crystals, further activating macrophages to release cytokines that increase immune cell recruitment in atherosclerosis 117. Intravenous ox-LDL-treated THP-1 cell-derived exosomes significantly worsened atherosclerosis in AS mice mainly due to miR-146a from THP-1-ox-LDL-exo, promoting the release of ROS and NETs by targeting SOD2 118.

Endothelial dysfunction is the main cause of atherosclerosis 119. The results of Cell Counting Kit-8 and test tube formation experiments showed that the growth and tube formation ability of endothelial cells were inhibited by the endocytosis of exosomes, suggesting that the exosomes produced by oxidized low-density lipoprotein-stimulated macrophages could reduce the growth and tube formation of endothelial cells 27.

Vascular smooth muscle cells migrate from the media to the intima, convert to a macrophage-like phenotype, and then engulf lipids to form foam cells, exacerbating atherosclerosis. Therefore, inhibition of VSMCs proliferation and migration can be used as a therapeutic target 120. Zhu et al. found that nicotine-stimulated macrophage exosomal miR-21-3p increases the migration and proliferation of vascular smooth muscle cells by targeting phosphatase and tension homology deleted on chromosome ten (PTEN), thus accelerating the development of atherosclerosis, revealing the partial role of nicotine in atherosclerosis 121. Niu et al. first demonstrated that EVs from foam cells might promote the migration and adhesion of VSMCs by integrating into VSMCs and activating ERK and Akt downstream 122. In addition, the migration of macrophages themselves also affects atherosclerosis. EV-derived miR-146a in atherosclerotic macrophages may accelerate the development of atherosclerosis by reducing the migration of macrophages and promoting their embedding in the vascular wall 123.

In addition to transporting miRNAs to mediate cell-to-cell communication, exosomes can contain many other biomolecules, such as proteins and long noncoding RNAs 20. Long noncoding RNA-growth arrest-specific 5 (lncRNA GAS5) associated with apoptosis factor caspase was found in ox-LDL-stimulated THP-1 macrophage exosomes and regulated apoptosis of macrophages and endothelial cells by internalization of exosomes. Therefore, inhibition of lncRNA GAS5 may be an effective approach for the treatment of atherosclerosis 124 (Table 1).

### Effects of exosomes from different sources internalized by macrophages

Similarly, macrophages can internalize other cell-derived exosomes and produce different effects. Several studies have shown that mesenchymal stem cell (MSC)-derived exosomes have the potential to reduce AS. For example, mesenchymal stem cell-derived exosomes miR-21a-5p and miR -let7 both mediate the polarization and infiltration of M2 macrophages to attenuate the progression of atherosclerosis in AS mice. MSC-derived exosomes containing miR-21A-5p promoted M2 polarization of RAW264.7 cells by inhibiting KLF6 expression and inhibited migration of RAW264.7 cells by inhibiting the ERK1/2 signaling pathway. The miR-let7/HMGA2/NF-κB pathway promoted the polarization of M2 macrophages in plaques, while the miR-let7/IGF2BP1/PTEN axis inhibited the infiltration of macrophages 128, 129. Therefore, MSC-derived exosomes are expected to be developed as a therapeutic agent for AS.

Dendritic cells are promoters of adaptive immunity, and exosomes derived from dendritic cells have been shown to be promising antitumor vaccines 130. Lin and colleagues isolated dendritic cell-derived exosomes and bone marrow-derived macrophages and established mouse models of AS, demonstrating that DCEX-derived miR-203-3p targets cathepsin S (Ctss) in bone marrow-derived macrophages to slow the progression of atherosclerosis at the cellular and mouse levels. The underlying molecular mechanism may involve the P38/MAPK signaling pathway 131.

Endothelial cells (ECs) are closely related to macrophages. Monocytes adhere to endothelial cells after activation, migrate to the subendothelial space, and then differentiate into macrophages, which play an important role in the pathogenesis of atherosclerosis 4. In human umbilical vein endothelial cells treated with ox-LDL, exosomal metastasis-associated pulmonary adenocarcinoma transcript 1 (MALAT1) was increased. Exosomes were cocultured with monocytes, resulting in the endocytosis of exosomes. Increased polarization of M2 macrophages was observed, and inhibition of MALAT1 expression in monocytes reversed exosome-mediated M2 macrophage polarization. A new mechanism of AS was revealed 132. Moreover, exosomes released by endothelial cells have been shown to enhance cholesterol efflux. One study used apoAI-expressing helper-dependent adenovirus (HDAd)-mediated arterial endothelial cells (ECs) to enhance cholesterol efflux in ECs *in vitro* and possibly upregulate cholesterol efflux pathways in other intima cell types. Based on this, they constructed an HDAd (HDAdXMoAntimiR33a5p) that transduced EC-derived exosomes and added macrophages or SMCs into the culture medium. Anti-miR-33a-5p was detected in these target cells, and the inhibition of miR-33a-5p on ABCA1 was relieved, which promotes cholesterol leakage 133. Another study found that stimulation of human coronary vascular endothelial cell (HCAEC)-derived EVs with ox-LDL enhanced lipid formation in human monocyte-derived macrophages (HMDMs), possibly due to EV-mediated upregulation of the Akt/NF-κB signaling pathway by miR-4306 134.

Obesity is a recognized risk factor for atherosclerosis, and exosomes from adipose tissue (AT) may be associated with metabolic complications of obesity 135-137. A study to evaluate the effects of AT exosomes on foam cell formation and macrophage polarization confirmed that visceral AT (VAT) exosomes promote atherosclerosis by regulating the formation and polarization of macrophage foam cells in obese mice induced by a high-fat diet (HFD), supporting the harm of obesity on AS 138. Yang et al. conducted an experiment based on the positive correlation between CagA-positive Helicobacter pylori (H. pylori) and atherosclerosis highlighted by seroepidemiological studies, revealing that exosomal CagA inhibited the expression of ABCA1 and ABCG1 by downregulating the expression of PPARγ and LXRα and promoted the formation of foam cells. This study verified the association between CagA-positive H. pylori infection and atherosclerosis, suggesting the contribution of prevention and eradication of CagA-positive Helicobacter pylori infection in reducing atherosclerosis 139-141.

Macrophage phenotypes are heterogeneous, and the regulation of exosomes on macrophage heterogeneity in plaques is also of interest. Exosome RNA was extracted and sequenced from the plasma of patients with chronic coronary artery disease (CAD) and healthy controls, and a series of bioinformatics analyses and experiments were performed to reveal that the intake of exosomes in CAD patients upregulated the expression of Ctss, Trem2, and Ccr2 mRNA in LPS-induced THP-1 cells 142. Another study confirmed the characteristic effect of EVs from ox-LDL-treated and/or KLF2-transduced ECs on monocyte/macrophage phenotypes *in vitro* and *in vivo* by regulating the expression of inflammation-related microRNA-155 in HUVECs. The above studies have revealed the influence of exosomes on the heterogeneity of macrophages 143 (Table 2).

### The diagnostic and therapeutic properties of macrophage exosomes in cardiovascular disease

Compared with traditional treatment, nanotechnology improves pharmacokinetic and chemical stability, reduces off-target effects, inhibits the progression of atherosclerosis, and targets macrophage imaging agent transfer and treatment to solve the treatment and diagnosis of atherosclerosis challenges (traditional anti-inflammatory therapy and the side effects of noninvasive identification of vulnerable plaque risk individuals), which provides a new strategy 29*.* However, as foreign substances, nanoparticles are bound to encounter a variety of physiological and cellular barriers from the injection site to their destination, which is designed to recognize and remove foreign substances. Therefore, it is very important to optimize the synthesis of nanoparticles to reduce their side effects and enhance their targeting. The development of bionic nanotechnology has made a certain contribution to solve these problems. Biological vectors that have been studied include membrane vectors, extracellular vesicle vectors, and viral vectors 31.

Exosomes consist of a variety of components, such as membrane proteins that ensure that they target specific sites, bilayer lipid membrane that protect biologically active components, metabolites, and nucleic acids that reflect the origin of their cells. Notably, exosomes can cross the blood-brain barrier (BBB) and can be used as drug carriers to target the brain. Additionally, exosomes have better physical and chemical properties and can be used as carriers for the targeted delivery of imaging agents and therapeutic molecules (including miRNA, siRNA, proteins, peptides, and chemotherapeutic drugs) 20. Macrophage-targeted delivery imaging agents may better identify individuals at risk of vulnerable plaques. In this regard, we summarize the imaging and treatment strategies based on exosome vectors.

Surface-functionalized exosomes can be imaged *in vivo* by a variety of methods (i.e., CT, magnetic resonance, fluorescence, and bioluminescence) for the study of various diseases. Chen and colleagues summarized the role of exosomes as imaging agents in glioma imaging, schizophrenia patients with nerve inflammation via *in vivo* imaging, neuroimaging in the body, and the application of mesenchymal stem cell-derived exosomes in MRI 31, 32, 34, 144-148. Noninvasive bioimaging techniques can help to display high-risk atherosclerotic plaques at the high spatial and temporal resolution, and the role of imaging agents cannot be ignored 29. Therefore, the design of exosome imaging contrast agents that can specifically target plaque macrophages can more accurately determine the biological characteristics of plaques and help quantify the burden of atherosclerosis and evaluate the effect of treatment. Many specific epitopes on the macrophage surface, such as S2P receptor, PS, mannose receptor, macrophage receptor MARCO, and LOX1, can be used to design exosome-based targeted imaging agents to improve imaging contrast 29.

In addition, minimally invasive liquid biopsy can be used to detect the composition of circulating exosomes for disease diagnosis and longitudinal progression tracking because exosomes carry various biological information, such as nucleic acids and proteins 20. The detection of exosomal miRNAs has shown great potential in cancer diagnosis and prognosis. Differential expression of miR-100 in the exosomes of tumor cells with oncogenic Kras contributes to the early diagnosis of cancer 149. The increase in miR-21 in circulating exosomes suggests malignant glioma, pancreatic cancer, colorectal cancer, liver cancer, breast cancer, ovarian cancer, and oesophageal cancer, while an increase in miR-21 in urine exosomes has been associated with bladder cancer and prostate cancer 150, 151. Similarly, enrichment of many miRNAs (miR-146a, miR-128, miR-185, miR-365, and miR-503) was found in macrophage-derived extracellular vesicles in atherosclerotic mouse models 123. Another study found that circulating exosomes in atherosclerotic patients were significantly higher than those in the control group. There were more leukocyte-derived exosomes 122. Both results suggested the potential diagnostic value of exosome detection in atherosclerosis.

Of note, in a study conducted by our team in 2021, we found that compared with plasmatic lncRNAs, exosomal lncRNAs showed more significant expression differences in large atherosclerotic stroke and combined detection of exo-lnc_000048, exo-lnc_001350 and exo-lnc_016442 has better diagnostic value. In addition, we found that exo-lnc_001350 and exo-lnc_016442 were better predictors of poor prognosis 152. Similar results were found in another study based on exosomal circRNA. Our experiment showed that exosomal circRNA could be used as a more efficient diagnostic marker for LAA stroke; much more interestingly, we found that exosomal circRNA could predict vulnerable plaques 153. Our studies have demonstrated the potential of exosomal non-coding RNAs as biomarkers. We are confident that macrophage-derived exosome noncoding RNA can also be a target for atherosclerotic biopsies in the future.

Moreover, exosomes associated with macrophages show great potential therapeutic value for atherosclerosis. Exosomes can be roughly divided into two strategies. (1) Exosomes can be used as drug carriers for target delivery to macrophages, which can be treat atherosclerosis through endocytosis. (2) Exosome inhibitors act on macrophage-derived exosomes to counter their effect on the progression of atherosclerosis. Wu and colleagues developed hexyl 5-aminolevulinate hydrochloride (HAL)-engineered M2 macrophage exosomes for the treatment of atherosclerosis, mainly through the release of anti-inflammatory cytokines to exert anti-inflammatory effects and the biosynthesis and metabolism of inherent heme, to produce anti-inflammatory carbon monoxide and bilirubin to further enhance anti-inflammatory effects 125. Furthermore, novel, efficient and less harmful treatments can be designed by combining exosomes with various biological behaviors in the development of atherosclerotic lesions as well as the abovementioned phenotypic transformations of cell proliferation, apoptosis and migration mediated by intercellular communication. In particular, different subtypes of macrophages have different or even opposite effects on plaque progression, so it is essential to design exosomes that target each subtype of macrophages for exogenous drug delivery. Although there are many studies on targeted therapy of exosomes, there are no reports on targeting macrophage subtypes. We will continue to pay close attention to this aspect in the future.

## Conclusion

The treatment of atherosclerosis should not be limited to lipid-lowering and traditional anti-inflammatory therapies, but more precise treatment plans are needed to reduce mortality and the incidence of adverse cardiovascular events. In this review, we have amply summarized the role of macrophages in the initiation and progression of atherosclerosis, the heterogeneity of their phenotype and distribution in plaques, and the different roles they play in atherosclerosis as therapeutic targets. In particular, we have summarized the role of macrophage-associated exosomes in atherosclerosis and the role of targeted macrophage-associated exosomes in the diagnosis and treatment of atherosclerosis, including minimally invasive liquid biopsies and biological imaging. We confidently believe that further research targeting macrophage exosomes will open up new perspectives for the diagnosis and treatment of atherosclerosis.

## Author Contributions

KYY drafted the manuscript. QX and MYN collected literatures. XDP and XYZ revised the manuscript. All authors read and approved the final manuscript.

## Figures and Tables

**Figure 4:**
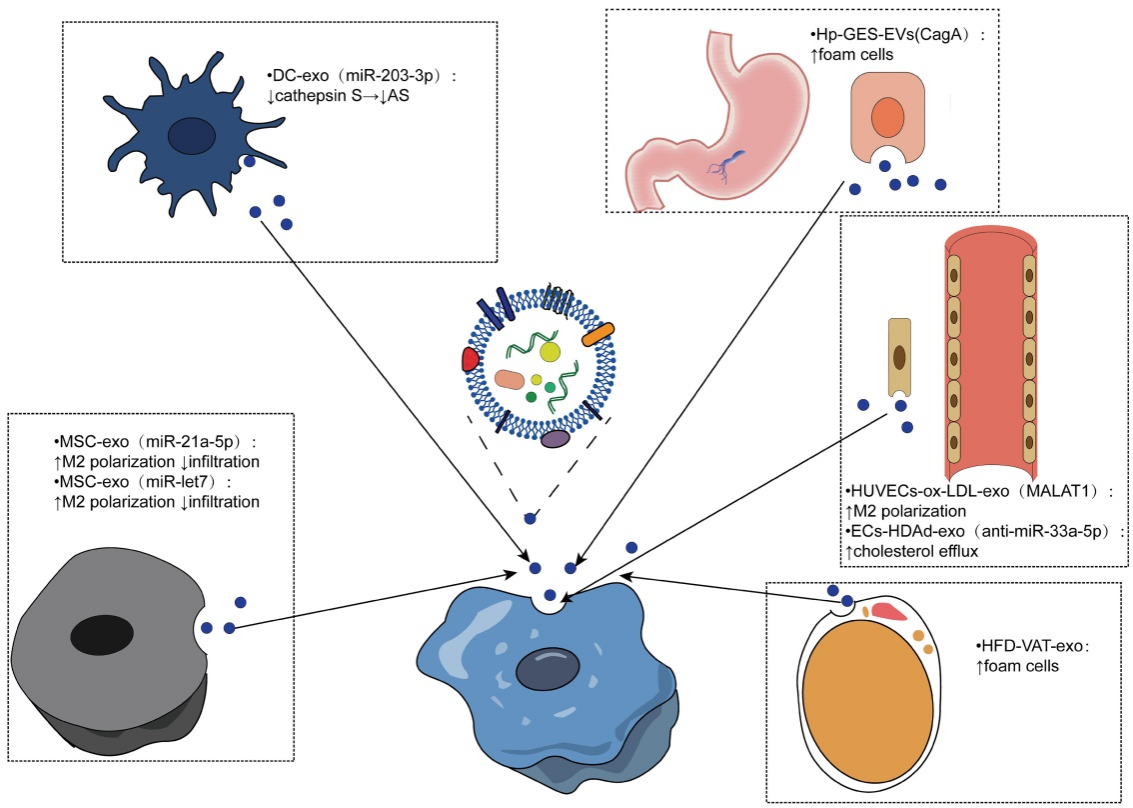
**Effects of exosomes from different sources being internalized by macrophages.** Exosomes from different cell sources mediate the intercellular communication with macrophages to realize the phenotypic transformation of macrophages, which is mainly reflected in the polarization and infiltration of macrophages, the formation of foam cells, and the outflow of cholesterol thus regulating the progression of atherosclerosis.

**Table 1:** Function of macrophage-derived exosomes and extracellular vesicles

exo/EVs	Contain	Mechanism	Function	Reference
BMDM- HG-exo	miR-486-5p		↑Hematopoietic; ↑Myeloid cells	109
BMDM-IL-4-exo	miRNA-99a/146b/378a	NF-κB and TNF-α signaling pathways	↓Hematopoietic; ↓Inflammation; ↑M2 polarization	101
THP1-IL-4-exo	miR-21/99a/146b/378a		↑Lipophagy; ↑Mitochondrial activity ↑Oxidative phosphorylation (OXPHOS); ↓Hematopoietic	113
THP-1-ox-LDL-exo	miR-106a-3p	CASP9 Caspase signaling pathway	↑VSMCs proliferation; ↓VSMCs apoptosis	115
Molecularly Engineered M2-exo	HAL		↓Inflammation Fluorescence imaging and tracking	125
THP-1-ox-LDL-exo	miR-146a	↑ROS	↑NETs→↑AS	118
M-ox-LDL-exo			↓ECs growth; ↓Tube formation	27
NM-exo	miR-21-3p	PTEN	↑VSMCs proliferation; ↑VSMCs migrate	121
THP-1-oxLDL-exo	lncRNA GAS5	caspase	↑ECs apoptosis; ↑Macrophage apoptosis	124
Foam cells-EVs		ERK, Akt	↑VSMCs adhesion; ↑VSMCs migrate	122
EVs	miR-503-5p	↓Smad 1,2,7	↓HCAEC proliferation; ↓Tube formation; ↑HCASMCs proliferation; ↑HCASMCs migrate	126
M-EVs	miR-185-3p	↓Smad 7	↑Lipid; ↑ECs adhesion; ↑ECs apoptosis; ↓ECs proliferation; ↑Inflammatory cytokines	127
M-EVs	miR-146a	IGF2BP1, HuR	↓Macrophage migrate	123

Akt: protein kinase B; CASP9: caspase 9; ECs: endothelial cells; ERK: extracellular signal-regulated kinase; EVs: extracellular vesicles; exo: exosomes; HAL: hexyl 5-aminolevulinate hydrochloride; HCAEC: human coronary vascular endothelial cell; HCASMCs: human coronary vascular smooth muscle cells; IGF2BP1: IGF2 mRNA binding protein 1; M: macrophage; NF-κB: nuclear factor-kappa b; TNF: tumor necrosis factor; Smad: sma- and mad-related proteins; VSMCs: vascular smooth muscle cells;

**Table 2:** Function of exosomes and extracellular vesicles from different cell sources on macrophages

exo/EVs	Contain	Mechanism	Function	Reference
MSC-exo	miR-21a-5p	KLF6 and ERK1/2 signaling pathways	↑M2 proliferation; ↓infiltration	128
	miR-let7	miR-let7/HMGA2/NF-κB miR-let7/IGF2BP1/PTEN	↑M2 proliferation ↓infiltration	129
DC-exo	miR-203-3p	↓cathepsin S	↓AS	131
Hp - GES- EVs	CagA	↓PPARγ and LXRα→ ↓cholesterol efflux	↑foam cells	139
HUVECs-ox-LDL-exo	MALAT1		↑M2 polarization	132
ECs-HDAd-exo	anti-miR-33a-5p		↑cholesterol efflux	133
HFD-VAT-exo		↓LXRα; ↓ABCA1 and ABCG1	↑foam cells	138
HCAEC-ox-LDL-EVs	miR-4306	↑Akt/ NF-κB signaling pathways	↑Lipid formation	134
ECs-ox-LDL/KLF2-EVs	miR-155		↑M2→M1	143

ABCA1: adenosine triphosphate binding cassette transporters 1; ABCG1: ATP-binding cassette transporter 8; AS: atherosclerosis; DC: dendritic cells; ERK: extracellular signal-regulated kinase; KLF: krüppel-like factor; LXRα: liver X receptor alpha; MSC: mesenchymal stem cell; PPARγ: peroxisome proliferator-activated receptor gamma;

## Abbreviations

ABC: adenosine triphosphate binding cassette transporters
ABCA1: adenosine triphosphate binding cassette transporters 1
Akt: protein kinase B
AMPK: AMP activated protein kinases
Apo: apolipoprotein
AS: atherosclerosis
ATF: activating transcription factors
Balb/C: balb 3t3 cells
C57BL/6: c57bl mice
CCL: c-c motif
CXCL: chemokine (C-X-C motif) ligand
CCR: receptors
CD: cluster of differentiation
CX_3_CR1: receptors
DCs: dendritic cells
exo: exosome
EVs: extracellular vehicles
ERK: extracellular signal-regulated kinase
HAL: hexyl 5-aminolevulinate hydrochloride
Hb: hemoglobin
HMOX-1: heme oxygenase-1
Hp: haptoglobin
IC: immune complexes
ICAM-1: intercellular adhesion molecule-1
IFN: interferon
IL: interleukin
Klf: Krüppel-like factor
LDL: low-density lipoproteins
LPS: lipopolysaccharide
M1: macrophages 1
M2: macrophage 2
MAPK: mitogen activated protein kinase
Mer TK: tyrosine kinase
MHC: major histocompatibility complex
MMP: matrix metalloproteinase
MR: mannose receptor
MVBs: multivesicular body
NF-κB: nuclear factor-kappa b
NLR: nucleotide-binding domain and leucine-rich repeat related
NLRP3: NLR family, pyrin domain containing 3
NM: nicotine-stimulated macrophage
Nrf2: NE-E2-related factor
PCSK9: proprotein convertase subtilisin/kexin Type 9
PPARγ: peroxisome proliferator-activated receptor gamma
RBC: red blood cell
ROS: reactive oxygen species
SMCs: smooth muscle cells
SNARE: soluble N-ethylmaleimide-sensitive-factor attachment protein receptor
ox-LDL: oxidized low density lipoprotein
SOD: superoxide dismutase
STAT: signal transducers and activators of transcription
Th: helper T cells
TGF: transforming growth factor
TLR: toll-like receptor
TNF: tumor necrosis factor
VCAM: vascular cell adhesion molecule
VSMCs: vascular smooth muscle cells

## References

[1] Li JJ, Fang CH (2004). Atheroscleritis is a more rational term for the pathological entity currently known as atherosclerosis. Medical hypotheses.

[2] Thompson RC, Allam AH, Lombardi GP, Wann LS, Sutherland ML, Sutherland JD (2013). Atherosclerosis across 4000 years of human history: the Horus study of four ancient populations. Lancet (London, England).

[3] Stary HC, Chandler AB, Dinsmore RE, Fuster V, Glagov S, Insull W Jr (1995). A definition of advanced types of atherosclerotic lesions and a histological classification of atherosclerosis. A report from the Committee on Vascular Lesions of the Council on Arteriosclerosis, American Heart Association. Circulation.

[4] Libby P, Buring JE, Badimon L, Hansson GK, Deanfield J, Bittencourt MS (2019). Atherosclerosis. Nature reviews Disease primers.

[5] Ross R (1999). Atherosclerosis-an inflammatory disease. The New England journal of medicine.

[6] Wick G, Xu Q (1999). Atherosclerosis-an autoimmune disease. Experimental gerontology.

[7] Soehnlein O, Libby P (2021). Targeting inflammation in atherosclerosis - from experimental insights to the clinic. Nature reviews Drug discovery.

[8] Montarello NJ, Nguyen MT, Wong DTL, Nicholls SJ, Psaltis PJ (2020). Inflammation in Coronary Atherosclerosis and Its Therapeutic Implications. Cardiovascular drugs and therapy.

[9] Lutgens E, Joffre J, van Os B, Ait-Oufella H (2021). Targeting cytokines and immune checkpoints in atherosclerosis with monoclonal antibodies. Atherosclerosis.

[10] Kim KW, Ivanov S, Williams JW (2020). Monocyte Recruitment, Specification, and Function in Atherosclerosis. Cells.

[11] Gisterå A, Hansson GK (2017). The immunology of atherosclerosis. Nature reviews Nephrology.

[12] Stary HC, Chandler AB, Glagov S, Guyton JR, Insull W Jr, Rosenfeld ME (1994). A definition of initial, fatty streak, and intermediate lesions of atherosclerosis. A report from the Committee on Vascular Lesions of the Council on Arteriosclerosis, American Heart Association. Circulation.

[13] Moroni F, Ammirati E, Norata GD, Magnoni M, Camici PG (2019). The Role of Monocytes and Macrophages in Human Atherosclerosis, Plaque Neoangiogenesis, and Atherothrombosis. Mediators of inflammation.

[14] Mills CD, Kincaid K, Alt JM, Heilman MJ, Hill AM (2000). M-1/M-2 macrophages and the Th1/Th2 paradigm. Journal of immunology (Baltimore, Md: 1950).

[15] Spitzer MH, Nolan GP (2016). Mass Cytometry: Single Cells, Many Features. Cell.

[16] Jinnouchi H, Guo L, Sakamoto A, Torii S, Sato Y, Cornelissen A (2020). Diversity of macrophage phenotypes and responses in atherosclerosis. Cellular and molecular life sciences: CMLS.

[17] Stöger JL, Gijbels MJ, van der Velden S, Manca M, van der Loos CM, Biessen EA (2012). Distribution of macrophage polarization markers in human atherosclerosis. Atherosclerosis.

[18] Pourcet B, Staels B (2018). Alternative macrophages in atherosclerosis: not always protective!. The Journal of clinical investigation.

[19] Moore KJ, Sheedy FJ, Fisher EA (2013). Macrophages in atherosclerosis: a dynamic balance. Nature reviews Immunology.

[20] Kalluri R, LeBleu VS (2020). The biology, function, and biomedical applications of exosomes. Science (New York, NY).

[21] Harada T, Yamamoto H, Kishida S, Kishida M, Awada C, Takao T (2017). Wnt5b-associated exosomes promote cancer cell migration and proliferation. Cancer science.

[22] Mineo M, Garfield SH, Taverna S, Flugy A, De Leo G, Alessandro R (2012). Exosomes released by K562 chronic myeloid leukemia cells promote angiogenesis in a Src-dependent fashion. Angiogenesis.

[23] Kalluri R (2016). The biology and function of exosomes in cancer. The Journal of clinical investigation.

[24] Gurunathan S, Kang MH, Kim JH (2021). A Comprehensive Review on Factors Influences Biogenesis, Functions, Therapeutic and Clinical Implications of Exosomes. International journal of nanomedicine.

[25] Shan X, Zhang C, Mai C, Hu X, Cheng N, Chen W (2021). The Biogenesis, Biological Functions, and Applications of Macrophage-Derived Exosomes. Frontiers in molecular biosciences.

[26] Liu S, Chen J, Shi J, Zhou W, Wang L, Fang W (2020). M1-like macrophage-derived exosomes suppress angiogenesis and exacerbate cardiac dysfunction in a myocardial infarction microenvironment. Basic research in cardiology.

[27] Huang C, Huang Y, Zhou Y, Nie W, Pu X, Xu X (2018). Exosomes derived from oxidized LDL-stimulated macrophages attenuate the growth and tube formation of endothelial cells. Molecular medicine reports.

[28] Funes SC, Rios M, Escobar-Vera J, Kalergis AM (2018). Implications of macrophage polarization in autoimmunity. Immunology.

[29] Chen W, Schilperoort M, Cao Y, Shi J, Tabas I, Tao W (2021). Macrophage-targeted nanomedicine for the diagnosis and treatment of atherosclerosis. Nature reviews Cardiology.

[30] Kim MS, Haney MJ, Zhao Y, Yuan D, Deygen I, Klyachko NL (2018). Engineering macrophage-derived exosomes for targeted paclitaxel delivery to pulmonary metastases: *in vitro* and *in vivo* evaluations. Nanomedicine: nanotechnology, biology, and medicine.

[31] Chen L, Hong W, Ren W, Xu T, Qian Z, He Z (2021). Recent progress in targeted delivery vectors based on biomimetic nanoparticles. Signal transduction and targeted therapy.

[32] Salunkhe S, Dheeraj, Basak M, Chitkara D, Mittal A (2020). Surface functionalization of exosomes for target-specific delivery and *in vivo* imaging & tracking: Strategies and significance. Journal of controlled release: official journal of the Controlled Release Society.

[33] Sun D, Zhuang X, Zhang S, Deng ZB, Grizzle W, Miller D (2013). Exosomes are endogenous nanoparticles that can deliver biological information between cells. Advanced drug delivery reviews.

[34] Lai CP, Mardini O, Ericsson M, Prabhakar S, Maguire C, Chen JW (2014). Dynamic biodistribution of extracellular vesicles *in vivo* using a multimodal imaging reporter. ACS nano.

[35] Kranzhöfer R, Browatzki M, Schmidt J, Kübler W (1999). Angiotensin II activates the proinflammatory transcription factor nuclear factor-kappaB in human monocytes. Biochemical and biophysical research communications.

[36] McMaster WG, Kirabo A, Madhur MS, Harrison DG (2015). Inflammation, immunity, and hypertensive end-organ damage. Circulation research.

[37] Mildner A, Schönheit J, Giladi A, David E, Lara-Astiaso D, Lorenzo-Vivas E (2017). Genomic Characterization of Murine Monocytes Reveals C/EBPβ Transcription Factor Dependence of Ly6C(-) Cells. Immunity.

[38] Tang N, Sun B, Gupta A, Rempel H, Pulliam L (2016). Monocyte exosomes induce adhesion molecules and cytokines via activation of NF-κB in endothelial cells. FASEB journal: official publication of the Federation of American Societies for Experimental Biology.

[39] Kennedy MA, Barrera GC, Nakamura K, Baldán A, Tarr P, Fishbein MC (2005). ABCG1 has a critical role in mediating cholesterol efflux to HDL and preventing cellular lipid accumulation. Cell metabolism.

[40] Wang N, Lan D, Chen W, Matsuura F, Tall AR (2004). ATP-binding cassette transporters G1 and G4 mediate cellular cholesterol efflux to high-density lipoproteins. Proceedings of the National Academy of Sciences of the United States of America.

[41] Tall AR, Yvan-Charvet L (2015). Cholesterol, inflammation and innate immunity. Nature reviews Immunology.

[42] Robbins CS, Hilgendorf I, Weber GF, Theurl I, Iwamoto Y, Figueiredo JL (2013). Local proliferation dominates lesional macrophage accumulation in atherosclerosis. Nature medicine.

[43] Swirski FK, Nahrendorf M, Libby P (2012). The ins and outs of inflammatory cells in atheromata. Cell metabolism.

[44] Bennett MR, Sinha S, Owens GK (2016). Vascular Smooth Muscle Cells in Atherosclerosis. Circulation research.

[45] Abilleira S, Bevan S, Markus HS (2006). The role of genetic variants of matrix metalloproteinases in coronary and carotid atherosclerosis. Journal of medical genetics.

[46] Geng YJ, Libby P (1995). Evidence for apoptosis in advanced human atheroma. Colocalization with interleukin-1 beta-converting enzyme. The American journal of pathology.

[47] Clarke MC, Talib S, Figg NL, Bennett MR (2010). Vascular smooth muscle cell apoptosis induces interleukin-1-directed inflammation: effects of hyperlipidemia-mediated inhibition of phagocytosis. Circulation research.

[48] Honold L, Nahrendorf M (2018). Resident and Monocyte-Derived Macrophages in Cardiovascular Disease. Circulation research.

[49] Nagenborg J, Goossens P, Biessen EAL, Donners M (2017). Heterogeneity of atherosclerotic plaque macrophage origin, phenotype and functions: Implications for treatment. European journal of pharmacology.

[50] Passlick B, Flieger D, Ziegler-Heitbrock HW (1989). Identification and characterization of a novel monocyte subpopulation in human peripheral blood. Blood.

[51] Ziegler-Heitbrock L, Ancuta P, Crowe S, Dalod M, Grau V, Hart DN (2010). Nomenclature of monocytes and dendritic cells in blood. Blood.

[52] Wong KL, Yeap WH, Tai JJ, Ong SM, Dang TM, Wong SC (2012). The three human monocyte subsets: implications for health and disease. Immunologic research.

[53] Gordon S (2003). Alternative activation of macrophages. Nature reviews Immunology.

[54] Swirski FK, Libby P, Aikawa E, Alcaide P, Luscinskas FW, Weissleder R (2007). Ly-6Chi monocytes dominate hypercholesterolemia-associated monocytosis and give rise to macrophages in atheromata. The Journal of clinical investigation.

[55] Chinetti-Gbaguidi G, Colin S, Staels B (2015). Macrophage subsets in atherosclerosis. Nature reviews Cardiology.

[56] Domschke G, Gleissner CA (2019). CXCL4-induced macrophages in human atherosclerosis. Cytokine.

[57] Murray PJ, Wynn TA (2011). Protective and pathogenic functions of macrophage subsets. Nature reviews Immunology.

[58] Mahdavian Delavary B, van der Veer WM, van Egmond M, Niessen FB, Beelen RH (2011). Macrophages in skin injury and repair. Immunobiology.

[59] Spencer M, Yao-Borengasser A, Unal R, Rasouli N, Gurley CM, Zhu B (2010). Adipose tissue macrophages in insulin-resistant subjects are associated with collagen VI and fibrosis and demonstrate alternative activation. American journal of physiology Endocrinology and metabolism.

[60] Mosser DM, Edwards JP (2008). Exploring the full spectrum of macrophage activation. Nature reviews Immunology.

[61] Barrett TJ (2020). Macrophages in Atherosclerosis Regression. Arteriosclerosis, thrombosis, and vascular biology.

[62] Colin S, Chinetti-Gbaguidi G, Staels B (2014). Macrophage phenotypes in atherosclerosis. Immunological reviews.

[63] Kadl A, Meher AK, Sharma PR, Lee MY, Doran AC, Johnstone SR (2010). Identification of a novel macrophage phenotype that develops in response to atherogenic phospholipids via Nrf2. Circulation research.

[64] Gleissner CA, Shaked I, Erbel C, Böckler D, Katus HA, Ley K (2010). CXCL4 downregulates the atheroprotective hemoglobin receptor CD163 in human macrophages. Circulation research.

[65] Finn AV, Nakano M, Polavarapu R, Karmali V, Saeed O, Zhao X (2012). Hemoglobin directs macrophage differentiation and prevents foam cell formation in human atherosclerotic plaques. Journal of the American College of Cardiology.

[66] Boyle JJ (2012). Heme and haemoglobin direct macrophage Mhem phenotype and counter foam cell formation in areas of intraplaque haemorrhage. Current opinion in lipidology.

[67] Cole JE, Park I, Ahern DJ, Kassiteridi C, Danso Abeam D, Goddard ME (2018). Immune cell census in murine atherosclerosis: cytometry by time of flight illuminates vascular myeloid cell diversity. Cardiovascular research.

[68] Zernecke A, Winkels H, Cochain C, Williams JW, Wolf D, Soehnlein O (2020). Meta-Analysis of Leukocyte Diversity in Atherosclerotic Mouse Aortas. Circulation research.

[69] Lim HY, Lim SY, Tan CK, Thiam CH, Goh CC, Carbajo D (2018). Hyaluronan Receptor LYVE-1-Expressing Macrophages Maintain Arterial Tone through Hyaluronan-Mediated Regulation of Smooth Muscle Cell Collagen. Immunity.

[70] Lin JD, Nishi H, Poles J, Niu X, McCauley C, Rahman K (2019). Single-cell analysis of fate-mapped macrophages reveals heterogeneity, including stem-like properties, during atherosclerosis progression and regression. JCI insight.

[71] Cochain C, Vafadarnejad E, Arampatzi P, Pelisek J, Winkels H, Ley K (2018). Single-Cell RNA-Seq Reveals the Transcriptional Landscape and Heterogeneity of Aortic Macrophages in Murine Atherosclerosis. Circulation research.

[72] Kim K, Shim D, Lee JS, Zaitsev K, Williams JW, Kim KW (2018). Transcriptome Analysis Reveals Nonfoamy Rather Than Foamy Plaque Macrophages Are Proinflammatory in Atherosclerotic Murine Models. Circulation research.

[73] Kim KW, Williams JW, Wang YT, Ivanov S, Gilfillan S, Colonna M (2016). MHC II+ resident peritoneal and pleural macrophages rely on IRF4 for development from circulating monocytes. The Journal of experimental medicine.

[74] Khallou-Laschet J, Varthaman A, Fornasa G, Compain C, Gaston AT, Clement M (2010). Macrophage plasticity in experimental atherosclerosis. PloS one.

[75] Peled M, Fisher EA (2014). Dynamic Aspects of Macrophage Polarization during Atherosclerosis Progression and Regression. Frontiers in immunology.

[76] Barlis P, Serruys PW, Devries A, Regar E (2008). Optical coherence tomography assessment of vulnerable plaque rupture: predilection for the plaque 'shoulder'. European heart journal.

[77] Gong M, Zhuo X, Ma A (2017). STAT6 Upregulation Promotes M2 Macrophage Polarization to Suppress Atherosclerosis. Medical science monitor basic research.

[78] Chinetti-Gbaguidi G, Baron M, Bouhlel MA, Vanhoutte J, Copin C, Sebti Y (2011). Human atherosclerotic plaque alternative macrophages display low cholesterol handling but high phagocytosis because of distinct activities of the PPARγ and LXRα pathways. Circulation research.

[79] Boyle JJ, Johns M, Lo J, Chiodini A, Ambrose N, Evans PC (2011). Heme induces heme oxygenase 1 via Nrf2: role in the homeostatic macrophage response to intraplaque hemorrhage. Arteriosclerosis, thrombosis, and vascular biology.

[80] Oram JF, Lawn RM, Garvin MR, Wade DP (2000). ABCA1 is the cAMP-inducible apolipoprotein receptor that mediates cholesterol secretion from macrophages. The Journal of biological chemistry.

[81] Chawla A, Boisvert WA, Lee CH, Laffitte BA, Barak Y, Joseph SB (2001). A PPAR gamma-LXR-ABCA1 pathway in macrophages is involved in cholesterol efflux and atherogenesis. Molecular cell.

[82] Raffai RL (2012). Apolipoprotein E regulation of myeloid cell plasticity in atherosclerosis. Current opinion in lipidology.

[83] Tangirala RK, Bischoff ED, Joseph SB, Wagner BL, Walczak R, Laffitte BA (2002). Identification of macrophage liver X receptors as inhibitors of atherosclerosis. Proceedings of the National Academy of Sciences of the United States of America.

[84] Naik SU, Wang X, Da Silva JS, Jaye M, Macphee CH, Reilly MP (2006). Pharmacological activation of liver X receptors promotes reverse cholesterol transport *in vivo*. Circulation.

[85] Libby P, Hansson GK (2019). From Focal Lipid Storage to Systemic Inflammation: JACC Review Topic of the Week. Journal of the American College of Cardiology.

[86] Libby P (2021). Inflammation in Atherosclerosis-No Longer a Theory. Clinical chemistry.

[87] Mayerl C, Lukasser M, Sedivy R, Niederegger H, Seiler R, Wick G (2006). Atherosclerosis research from past to present-on the track of two pathologists with opposing views, Carl von Rokitansky and Rudolf Virchow. Virchows Archiv: an international journal of pathology.

[88] Ridker PM, Everett BM, Thuren T, MacFadyen JG, Chang WH, Ballantyne C (2017). Antiinflammatory Therapy with Canakinumab for Atherosclerotic Disease. The New England journal of medicine.

[89] Tardif JC, Kouz S, Waters DD, Bertrand OF, Diaz R, Maggioni AP (2019). Efficacy and Safety of Low-Dose Colchicine after Myocardial Infarction. The New England journal of medicine.

[90] Lusis AJ (2000). Atherosclerosis. Nature.

[91] Ramji DP, Davies TS (2015). Cytokines in atherosclerosis: Key players in all stages of disease and promising therapeutic targets. Cytokine & growth factor reviews.

[92] Roy P, Orecchioni M, Ley K (2021). How the immune system shapes atherosclerosis: roles of innate and adaptive immunity. Nature reviews Immunology.

[93] Tabas I (2005). Consequences and therapeutic implications of macrophage apoptosis in atherosclerosis: the importance of lesion stage and phagocytic efficiency. Arteriosclerosis, thrombosis, and vascular biology.

[94] Cai B, Thorp EB, Doran AC, Sansbury BE, Daemen MJ, Dorweiler B (2017). MerTK receptor cleavage promotes plaque necrosis and defective resolution in atherosclerosis. The Journal of clinical investigation.

[95] Tajbakhsh A, Rezaee M, Kovanen PT, Sahebkar A (2018). Efferocytosis in atherosclerotic lesions: Malfunctioning regulatory pathways and control mechanisms. Pharmacology & therapeutics.

[96] Basatemur GL, Jørgensen HF, Clarke MCH, Bennett MR, Mallat Z (2019). Vascular smooth muscle cells in atherosclerosis. Nature reviews Cardiology.

[97] Zhang Y, McCluskey K, Fujii K, Wahl LM (1998). Differential regulation of monocyte matrix metalloproteinase and TIMP-1 production by TNF-alpha, granulocyte-macrophage CSF, and IL-1 beta through prostaglandin-dependent and -independent mechanisms. Journal of immunology (Baltimore, Md: 1950).

[98] Burgmaier M, Milzi A, Dettori R, Burgmaier K, Marx N, Reith S (2018). Co-localization of plaque macrophages with calcification is associated with a more vulnerable plaque phenotype and a greater calcification burden in coronary target segments as determined by OCT. PloS one.

[99] Reith S, Milzi A, Dettori R, Marx N, Burgmaier M (2018). Predictors for target lesion microcalcifications in patients with stable coronary artery disease: an optical coherence tomography study. Clinical research in cardiology: official journal of the German Cardiac Society.

[100] Shioi A, Ikari Y (2018). Plaque Calcification During Atherosclerosis Progression and Regression. Journal of atherosclerosis and thrombosis.

[101] Bouchareychas L, Duong P, Covarrubias S, Alsop E, Phu TA, Chung A (2020). Macrophage Exosomes Resolve Atherosclerosis by Regulating Hematopoiesis and Inflammation via MicroRNA Cargo. Cell reports.

[102] Théry C, Zitvogel L, Amigorena S (2002). Exosomes: composition, biogenesis and function. Nature reviews Immunology.

[103] Gould SJ, Raposo G (2013). As we wait: coping with an imperfect nomenclature for extracellular vesicles. Journal of extracellular vesicles.

[104] Kowal J, Arras G, Colombo M, Jouve M, Morath JP, Primdal-Bengtson B (2016). Proteomic comparison defines novel markers to characterize heterogeneous populations of extracellular vesicle subtypes. Proceedings of the National Academy of Sciences of the United States of America.

[105] Li ZG, Scott MJ, Brzóska T, Sundd P, Li YH, Billiar TR (2018). Lung epithelial cell-derived IL-25 negatively regulates LPS-induced exosome release from macrophages. Military Medical Research.

[106] Ibáñez F, Montesinos J, Ureña-Peralta JR, Guerri C, Pascual M (2019). TLR4 participates in the transmission of ethanol-induced neuroinflammation via astrocyte-derived extracellular vesicles. Journal of neuroinflammation.

[107] Munagala R, Aqil F, Jeyabalan J, Gupta RC (2016). Bovine milk-derived exosomes for drug delivery. Cancer letters.

[108] Gurung S, Perocheau D, Touramanidou L, Baruteau J (2021). The exosome journey: from biogenesis to uptake and intracellular signalling. Cell communication and signaling: CCS.

[109] Bouchareychas L, Duong P, Phu TA, Alsop E, Meechoovet B, Reiman R (2021). High glucose macrophage exosomes enhance atherosclerosis by driving cellular proliferation & hematopoiesis. iScience.

[110] Wang X, Collins HL, Ranalletta M, Fuki IV, Billheimer JT, Rothblat GH (2007). Macrophage ABCA1 and ABCG1, but not SR-BI, promote macrophage reverse cholesterol transport *in vivo*. The Journal of clinical investigation.

[111] Yvan-Charvet L, Pagler T, Gautier EL, Avagyan S, Siry RL, Han S (2010). ATP-binding cassette transporters and HDL suppress hematopoietic stem cell proliferation. Science (New York, NY).

[112] Groenen AG, Halmos B, Tall AR, Westerterp M (2021). Cholesterol efflux pathways, inflammation, and atherosclerosis. Critical reviews in biochemistry and molecular biology.

[113] Phu TA, Ng M, Vu NK, Bouchareychas L, Raffai RL (2022). IL-4 polarized human macrophage exosomes control cardiometabolic inflammation & diabetes in obesity. Molecular therapy: the journal of the American Society of Gene Therapy.

[114] Kuida K (2000). Caspase-9. The international journal of biochemistry & cell biology.

[115] Liu Y, Zhang WL, Gu JJ, Sun YQ, Cui HZ, Bu JQ (2020). Exosome-mediated miR-106a-3p derived from ox-LDL exposed macrophages accelerated cell proliferation and repressed cell apoptosis of human vascular smooth muscle cells. European review for medical and pharmacological sciences.

[116] Branzk N, Lubojemska A, Hardison SE, Wang Q, Gutierrez MG, Brown GD (2014). Neutrophils sense microbe size and selectively release neutrophil extracellular traps in response to large pathogens. Nature immunology.

[117] Warnatsch A, Ioannou M, Wang Q, Papayannopoulos V (2015). Inflammation. Neutrophil extracellular traps license macrophages for cytokine production in atherosclerosis. Science (New York, NY).

[118] Zhang YG, Song Y, Guo XL, Miao RY, Fu YQ, Miao CF (2019). Exosomes derived from oxLDL-stimulated macrophages induce neutrophil extracellular traps to drive atherosclerosis. Cell cycle (Georgetown, Tex).

[119] Eren E, Yilmaz N, Aydin O (2013). Functionally defective high-density lipoprotein and paraoxonase: a couple for endothelial dysfunction in atherosclerosis. Cholesterol.

[120] Weinert S, Poitz DM, Auffermann-Gretzinger S, Eger L, Herold J, Medunjanin S (2013). The lysosomal transfer of LDL/cholesterol from macrophages into vascular smooth muscle cells induces their phenotypic alteration. Cardiovascular research.

[121] Zhu J, Liu B, Wang Z, Wang D, Ni H, Zhang L (2019). Exosomes from nicotine-stimulated macrophages accelerate atherosclerosis through miR-21-3p/PTEN-mediated VSMC migration and proliferation. Theranostics.

[122] Niu C, Wang X, Zhao M, Cai T, Liu P, Li J (2016). Macrophage Foam Cell-Derived Extracellular Vesicles Promote Vascular Smooth Muscle Cell Migration and Adhesion. Journal of the American Heart Association.

[123] Nguyen MA, Karunakaran D, Geoffrion M, Cheng HS, Tandoc K, Perisic Matic L (2018). Extracellular Vesicles Secreted by Atherogenic Macrophages Transfer MicroRNA to Inhibit Cell Migration. Arteriosclerosis, thrombosis, and vascular biology.

[124] Chen L, Yang W, Guo Y, Chen W, Zheng P, Zeng J (2017). Exosomal lncRNA GAS5 regulates the apoptosis of macrophages and vascular endothelial cells in atherosclerosis. PloS one.

[125] Wu G, Zhang J, Zhao Q, Zhuang W, Ding J, Zhang C (2020). Molecularly Engineered Macrophage-Derived Exosomes with Inflammation Tropism and Intrinsic Heme Biosynthesis for Atherosclerosis Treatment. Angewandte Chemie (International ed in English).

[126] Wang Y, Xu Z, Wang X, Zheng J, Peng L, Zhou Y (2021). Extracellular-vesicle containing miRNA-503-5p released by macrophages contributes to atherosclerosis. Aging.

[127] Li K, Cui M, Zhang K, Wang G, Zhai S (2021). M1 macrophages-derived extracellular vesicles elevate microRNA-185-3p to aggravate the development of atherosclerosis in ApoE(-/-) mice by inhibiting small mothers against decapentaplegic 7. International immunopharmacology.

[128] Ma J, Chen L, Zhu X, Li Q, Hu L, Li H (2021). Mesenchymal stem cell-derived exosomal miR-21a-5p promotes M2 macrophage polarization and reduces macrophage infiltration to attenuate atherosclerosis. Acta biochimica et biophysica Sinica.

[129] Li J, Xue H, Li T, Chu X, Xin D, Xiong Y (2019). Exosomes derived from mesenchymal stem cells attenuate the progression of atherosclerosis in ApoE(-/-) mice via miR-let7 mediated infiltration and polarization of M2 macrophage. Biochemical and biophysical research communications.

[130] Pitt JM, Charrier M, Viaud S, André F, Besse B, Chaput N (2014). Dendritic cell-derived exosomes as immunotherapies in the fight against cancer. Journal of immunology (Baltimore, Md: 1950).

[131] Lin B, Xie W, Zeng C, Wu X, Chen A, Li H (2021). Transfer of exosomal microRNA-203-3p from dendritic cells to bone marrow-derived macrophages reduces development of atherosclerosis by downregulating Ctss in mice. Aging.

[132] Huang C, Han J, Wu Y, Li S, Wang Q, Lin W (2018). Exosomal MALAT1 derived from oxidized low-density lipoprotein-treated endothelial cells promotes M2 macrophage polarization. Molecular medicine reports.

[133] Stamatikos A, Knight E, Vojtech L, Bi L, Wacker BK, Tang C (2020). Exosome-Mediated Transfer of Anti-miR-33a-5p from Transduced Endothelial Cells Enhances Macrophage and Vascular Smooth Muscle Cell Cholesterol Efflux. Human gene therapy.

[134] Yang Y, Luo H, Zhou C, Zhang R, Liu S, Zhu X (2019). Regulation of capillary tubules and lipid formation in vascular endothelial cells and macrophages via extracellular vesicle-mediated microRNA-4306 transfer. The Journal of international medical research.

[135] Van Gaal LF, Mertens IL, De Block CE (2006). Mechanisms linking obesity with cardiovascular disease. Nature.

[136] Kataoka Y, Hammadah M, Puri R, Duggal B, Uno K, Kapadia SR (2015). Plaque vulnerability at non-culprit lesions in obese patients with coronary artery disease: Frequency-domain optical coherence tomography analysis. European journal of preventive cardiology.

[137] Yonetsu T, Kato K, Uemura S, Kim BK, Jang Y, Kang SJ (2013). Features of coronary plaque in patients with metabolic syndrome and diabetes mellitus assessed by 3-vessel optical coherence tomography. Circulation Cardiovascular imaging.

[138] Xie Z, Wang X, Liu X, Du H, Sun C, Shao X (2018). Adipose-Derived Exosomes Exert Proatherogenic Effects by Regulating Macrophage Foam Cell Formation and Polarization. Journal of the American Heart Association.

[139] Yang S, Xia YP, Luo XY, Chen SL, Li BW, Ye ZM (2019). Exosomal CagA derived from Helicobacter pylori-infected gastric epithelial cells induces macrophage foam cell formation and promotes atherosclerosis. Journal of molecular and cellular cardiology.

[140] Elkind MS, Luna JM, Moon YP, Boden-Albala B, Liu KM, Spitalnik S (2010). Infectious burden and carotid plaque thickness: the northern Manhattan study. Stroke.

[141] Liu J, Wang F, Shi S (2015). Helicobacter pylori Infection Increase the Risk of Myocardial Infarction: A Meta-Analysis of 26 Studies Involving more than 20,000 Participants. Helicobacter.

[142] Li X, He X, Wang J, Wang D, Cong P, Zhu A (2020). The Regulation of Exosome-Derived miRNA on Heterogeneity of Macrophages in Atherosclerotic Plaques. Frontiers in immunology.

[143] He S, Wu C, Xiao J, Li D, Sun Z, Li M (2018). Endothelial extracellular vesicles modulate the macrophage phenotype: Potential implications in atherosclerosis. Scandinavian journal of immunology.

[144] Pasternak O, Kubicki M, Shenton ME (2016). *In vivo* imaging of neuroinflammation in schizophrenia. Schizophrenia research.

[145] Jia G, Han Y, An Y, Ding Y, He C, Wang X (2018). NRP-1 targeted and cargo-loaded exosomes facilitate simultaneous imaging and therapy of glioma *in vitro* and *in vivo*. Biomaterials.

[146] Betzer O, Perets N, Angel A, Motiei M, Sadan T, Yadid G (2017). *In vivo* Neuroimaging of Exosomes Using Gold Nanoparticles. ACS nano.

[147] Busato A, Bonafede R, Bontempi P, Scambi I, Schiaffino L, Benati D (2016). Magnetic resonance imaging of ultrasmall superparamagnetic iron oxide-labeled exosomes from stem cells: a new method to obtain labeled exosomes. International journal of nanomedicine.

[148] Liu T, Zhu Y, Zhao R, Wei X, Xin X (2020). Visualization of exosomes from mesenchymal stem cells *in vivo* by magnetic resonance imaging. Magnetic resonance imaging.

[149] Cha DJ, Franklin JL, Dou Y, Liu Q, Higginbotham JN, Demory Beckler M (2015). KRAS-dependent sorting of miRNA to exosomes. eLife.

[150] Salehi M, Sharifi M (2018). Exosomal miRNAs as novel cancer biomarkers: Challenges and opportunities. Journal of cellular physiology.

[151] Thind A, Wilson C (2016). Exosomal miRNAs as cancer biomarkers and therapeutic targets. Journal of extracellular vesicles.

[152] Zhang S, Wang X, Yin R, Xiao Q, Ding Y, Zhu X (2021). Circulating exosomal lncRNAs as predictors of risk and unfavorable prognosis for large artery atherosclerotic stroke. Clinical and translational medicine.

[153] Xiao Q, Hou R, Li H, Zhang S, Zhang F, Zhu X (2022). Circulating Exosomal circRNAs Contribute to Potential Diagnostic Value of Large Artery Atherosclerotic Stroke. Frontiers in immunology.

